# Family Peace of Mind of Individuals Over 65 Years of Age With Chronic Diseases: A Cross‐Sectional Study

**DOI:** 10.1111/jep.70063

**Published:** 2025-03-26

**Authors:** Emre Birinci, Ceyda Bayram, Ayşe Özkaraman

**Affiliations:** ^1^ Ageing Studies Application and Research Unit Anadolu University Eskişehir Türkiye; ^2^ Department of Nursing, Faculty of Health Science Eskişehir Osmangazi University Eskişehir Türkiye

**Keywords:** aged, chronic diseases, dependents, family peace, self‐efficacy

## Abstract

**Objective:**

The purpose of this study was to assess the level of family peace among people over 65 who have chronic diseases and to look at the level of peace in relation to specific factors.

**Method:**

This cross‐sectional study was conducted between 1 July and 31 October 2023 in Internal Medicine policlinics and clinics of a hospital in Eskişehir/Türkiye. The data were collected using the ‘Introduction Form’, ‘Self‐Efficacy Scale’ and ‘Family Peace Scale’. While the data were given in the form of number, percentage, mean and percantage, Mann–Whitney *U* and Kruskal–Wallis *T* tests and Spearman's correlation analysis were applied in statistical analysis.

**Results:**

The total mean score of the family peace scale was 64.82 ± 9.041, the deep wear sub‐dimension score was 34.11 ± 5.66, and the general contentment sub‐dimension score was 30.71 ± 4.64. Age of the patients showed a significantly favourable association with the overall family peace score (*r* = 0.149; *p* = 0.019) (Table 4). Individual with dependents had statistically significantly lower total and sub‐dimension scores of family peace (*p* = 0.004, *p* = 0.030, *p* = 0.007, respectively). It was found that family peace increased significantly in the positive direction as the level of self‐efficacy of the patients increased (*r* = 0.389, *p* < 0.001).

**Conclusion:**

Family peace of mind is strongly associated with age, dependents and self‐efficacy perceptions of individuals.

## 1 Introduction

Peace of mind is a state of inner comfort, calmness, serenity and well‐being [1]. In order to maintain peace of mind, the harmony of relationships at all levels ranging from nations to groups, families, couples and friends is necessary [2]. Marriage, family, friend groups, work environment and social spaces are environments that provide information about interpersonal peace [3]. In order to ensure interpersonal peace, the focus is on harmony and peace in all relationships in daily life [2, 4].

Interpersonal peace in the family environment is used when a family is healthy, happy or successful in daily life [5]. Family peace varies according to the life cycle, members entering and leaving the family, changing responsibilities of family members [6] and health, social, economic and cultural situation [4, 7, 8]. Family peace can be affected by negative experiences that indicate deep wear and tear such as low income, divorce, substance abuse, violence, stress; positive emotions and behaviours such as understanding the other individual, cooperation, calmness, peaceful environment, problem solving, communication skills, compassion, optimism; the general mood of the family and the health status of family members [4, 8]. It is also emphasised that age and gender affect family peace, and older individuals contribute more to the peaceful environment than young people based on their wisdom and life experiences [9].

The presence of chronic diseases among family members is one of the factors that negatively affect the family environment [10]. Chronic diseases can lead to physical, mental [11, 12], economic and social problems, disturbance of peace in the family [13], and changes in lifestyles of the patient and caregiver family members [12, 14]. In addition, it is known that the social and family life of the patient with sufficient self‐care power is less affected [15].

Studies provide results on the care burden, physiological–psychological health problems, social and economic status of family members caring for the elderly and individuals with chronic diseases. In addition, there are also findings on concepts such as happiness, fun, comfort and well‐being in the elderly [7, 16, 17, 18, 19]. However, in the context of health services, there are gaps in the literature on family peace of older people with chronic diseases. The purpose of this study was to assess the level of family peace of individuals over the age of 65 with chronic diseases and to examine the level of family peace in terms of certain variables. The research questions are as follows:

- What is the level of family peace of individuals over the age of 65 who suffer from chronic diseases?
- Are there factors that affect the family peace of individuals over the age of 65 who suffer from chronic diseases?
- What are the factors affecting the family peace of individuals over the age of 65 who suffer from chronic diseases?

## 2 Methods

### 2.1 Design, Setting and Participants

**Figure 1:**
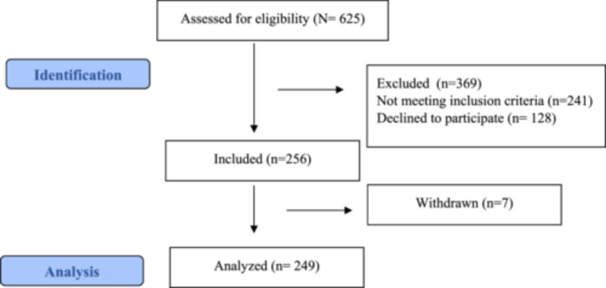
Flow diagram of the study.

This cross‐sectional study was reported according to STrengthening the Reporting of OBservational studies in Epidemiology (STROBE) Statement on cross‐sectional studies. Recruitment of this study was conducted in Internal Medicine outpatient clinics and clinics of a hospital in Eskisehir/Türkiye between 1 July and 31 October 2023. Individuals were selected through convenience sampling according to inclusion criteria: being treated and diagnosed with the chronic disease, to agree to participate in the study, and to be literate in Turkish. Exclusion criteria were being under 65 years of age, living alone, and requesting to withdraw from the study (Figure 1). In the study, no calculation was made when determining the sample number, and the complete count method was used to include all patients who satisfied the inclusion criteria in the sample between 1 May 2023 and 31 October 2023.

### 2.2 Study Variables

Family peace comprised deep attrition and general contentment. The present study set out to analyse the effect of socio‐demographic and medical characteristics, as well as self‐efficacy status obtained from self‐assessment of individuals, on the level of family peace.

### 2.3 Measurement Tools

#### 2.3.1 Introduction Form

This form was prepared by the researchers to collect information about some socio‐demographic characteristics (age, gender, marital status, educational status, economic status, dependents) and disease status (chronic disease status, medications taken, harmful habits, caregiver).

#### 2.3.2 Self‐Efficacy Scale

The scale was developed by Sherer and colleagues in 1982 [20] and adapted into Turkish by Gözüm and Aksayan in 1999. The Cronbach *⍺* internal consistency coefficient of the Turkish version of the scale is 0.81. The scale is a 5‐point Likert‐type self‐assessment tool consisting of 23 items. The scores given for items 1, 3, 8, 9, 13, 15, 19, 21 and 23 in the scale are taken as basis. Items 2, 4, 5, 6, 7, 10, 11, 12, 14, 16, 17, 18, 20 and 22 are scored in the opposite direction. The scale has a minimum of 23 and a maximum of 115 points. An elevated aggregate score signifies that the person's assessment of summarisedness is at an acceptable degree [21].

#### 2.3.3 Family Peace Scale

This scale was developed by Özdemir and Bakiler in 2021 to measure peace within the family [5]. It is a 5‐point Likert‐type scale consisting of 15 questions to collect information about peace in the family. The scale has an internal consistency coefficient of 0.95, and the averages of the lower and upper dimensions have correlations between 0.69 and 0.82 (*p* < 0.01). The scale has two sub‐factors, namely, deep attrition and general contentment. The total score is calculated by reverse scoring the items between 8 and 15 in the scale. The higher the scores obtained from the scale, the higher the family peace [22].

### 2.4 Data Collection

Face‐to‐face interviews were used to gather research data. Individuals in the outpatient clinic and clinic between 8 and 17 h were informed about the research by the researchers and invited to participate in the study. In the clinic's patient rooms and in the outpatient clinic's quiet training area, participants in the study completed data collecting forms. Individuals completed the forms independently; the researchers did not intervene during this process. The completed forms were received by the researcher. The completion of the data collection forms took ~20 min. The information obtained from the forms was analysed by a statistical expert independent of the researchers.

### 2.5 Data Analysis

The data obtained from the study were analysed using IBM Statistical Package for Social Science (SPSS) 27.0 package programme. The analyses were conducted by an independent statistician who was not involved in the research. For qualitative variables, the summary values were expressed as percentage and frequency, whereas for quantitative data, the expression was as mean and quarterly. The appropriateness of the data for a normal distribution was examined using the Shapiro–Wilk test. When comparing groups that did not exhibit a normal distribution, the Mann–Whitney *U* and Kruskal–Wallis *T* tests were utilised. Correlation distributions of non‐normally distributed data were analysed by Spearman's correlation test. Significance was accepted as *p* < 0.05 in statistical evaluation.

### 2.6 Ethical Considerations

Before starting the study, the ethical suitability of the research was examined by the relevant ethics committee and approved in writing on 02.05.2023 with the number 522553. Written consent was received from the relevant institution after the relevant ethics committee gave its approval. Written informed permission was obtained from the persons who would be involved in the study after they were informed about it. Permission was obtained from Sebahat Gözüm for the use of the Self‐Efficacy Scale and Pınar Özdemir for the Family Peace Scale.

## 3 Results

The mean age of the individuals in the study was 73.38 ± 6.78 years. The total mean score of the family peace scale was 64.82 ± 9.041, the deep wear sub‐dimension score was 34.11 ± 5.66, and the general contentment sub‐dimension score was 30.71 ± 4.64. The total score of the self‐efficacy scale was 83.92 ± 12.27 (Table 1).

**Table 1:** Distribution of patients' age, family peace scale and self‐efficacy scale scores (*n* = 249).

Variables		ort ± sd	Min–Max
Age (year)		73.38 ± 6.78	65–101
Family peace scale	The deep wear	34.11 ± 5.66	8–40
	The general contentment	30.71 ± 4.64	12–35
	Total	64.82 ± 9.041	29–75
Self efficacy scale total		83.92 ± 12.27	41–106

Among the individuals, 55% were female, 53.8% were married, 38.6% were elementary school graduates, and 71.5% had an income equivalent to their expenses. Whilst the majority of the individuals were part of a nuclear family unit, 17.7% were providing care for their relatives. Total and sub‐dimension scores of the family peace scale were not statistically different according to gender, marital status, educational status, income status, chronic disease status, harmful habits and the presence of caregivers (*p *> 0.05). It was found that the total and sub‐dimension scores of family peace were significantly lower in those who were responsible for the care of their relatives compared to the others (*p* = 0.004, *p* = 0.030, *p* = 0.007, respectively) (Table 2).

**Table 2:** Distribution of total and subscale mean scores of the family peace scale according to socio‐demographic characteristics of patients (*n* = 249).

Variables			The deep wear	Test/*p*	The general contentment	Test/*p*	Total	Test/*p*
Gender	*N*	%	Mean (Q1–Q3)	*U*	Mean (Q1–Q3)	*U*	Mean (Q1–Q3)	*U*
Female	137	55	35 (32–38)	7055.5	32 (28–35)	7492	67 (61–71)	7145
Male	112	45	35 (32–37.75)	0.273	32 (28–35)	0.746	66.5 (61.25–70.75)	0.350
Marital status			Mean (Q1–Q3)	*U*	Mean (Q1–Q3)	*U*	Mean (Q1–Q3)	*U*
Married	134	53.8	35 (32–38)	7652	33 (28–35)	6677	67 (62–71)	7300.5
Single	115	46.2	35 (32–38)	0.925	31 (28–34)	0.065	66 (61–71)	0.474
Education status			Mean (Q1–Q3)	*U*	Mean (Q1–Q3)	*U*	Mean (Q1–Q3)	KW
Literate	38	15.3	35 (32.75–37.75)	2.407	32.5 (28–35)	9.425	67 (61–71)	7.224
Elementary school	96	38.6	35.5 (32–39)	0.661	33 (30–35)	0.051	68 (62–71.75)	0.125
Middle school	48	19.3	34 (32–37)		30 (28–34)		64 (61–69)	
High school	54	21.7	35 (31.75–38)		31.5 (27.5–31.5)		66 (60.75–70.25)	
University	13	5.2	34 (31–38)		28 (27–34.5)		63 (58.5–70)	
Level of income			Mean (Q1–Q3)	KW	Mean (Q1–Q3)	KW	Mean (Q1–Q3)	KW
Income < expenses	14	5.6	35 (26.525–40)	0.565	34.5 (28–35)	2.004	63.5 (59.25–74.25)	0.120
Income = expenses	178	71.5	35 (32–38)	0.754	32 (28–35)	0.367	67 (61.75–71)	0.942
Income > expenses	57	22.9	35 (32–37)		33 (28–35)		67 (60–70.5)	
Family type			Mean (Q1–Q3)	*U*	Mean (Q1–Q3)	*U*	Mean (Q1–Q3)	*U*
Nuclear	217	87.1	35 (32–38)	3302.5	32 (28–35)	3251	67 (61–71)	3347.5
Extended	32	12.9	34.5 (31–38)	0.654	33 (27.25–35)	0.555	68 (60–72.5)	0.743
Caregiver			Mean (Q1–Q3)	*U*	Mean (Q1–Q3)	*U*	Mean (Q1–Q3)	*U*
Yes	44	17.7	34 (30–35.75)	3253.5	29.5 (27–34)	3585.5	63 (57.25–68.75)	3337
No	205	82.3	35 (32–38)	0.004	33 (28–35)	0.030	67 (62–71)	0.007

Abbreviations: KW, Kruskal–Wallis test; *U*, Mann–Whitney *U* test.

When the sub‐dimension and overall scores of the groups with various chronic illnesses were compared, it was determined that there was no statistically significant difference (*p* > 0.05). In addition, the differences between those with and without harmful behaviours were also analysed, and patients with harmful behaviours had a higher mean score in the ‘The deep wear’ sub‐dimension compared to those without (36 vs. 35); nonetheless, indicates that this difference was not statistically significant (*p* = 0.0541). There was no statistically significant difference between the sub‐dimension and total ratings based on the presence of caregivers (*p* > 0.05) (Table 3).

**Table 3:** Distribution of sub‐dimension and total mean scores of the family peace scale according to the medical characteristics of the patients (*n* = 249).

Variables			The deep wear	Test/*p*	The general contentment	Test/*p*	Total	Test/*p*
Type of chronic disease	*n*	**%**	Mean (Q1–Q3)	KW	Mean (Q1–Q3)	KW	Mean (Q1–Q3)	KW
Chronic lung diseases^a^	69	27.71	34 (31–37)	7.494	32 (28–34)	3876	66 (60.5–71)	3.271
Heart diseases^b^	31	12.44	35 (32–36)	0.379	33 (28–35)	0.796	65 (59–71)	0.859
Diabetes mellitus	46	18.45	36 (32–38.25)		31.5 (28–35)		66 (61–71)	
Cerebrovascular diseases	5	2	33 (33–36.5)		30 (30–35)		63 (63–71.50)	
Hypertension	42	17	36 (32–39)		33 (27.75–35)		68.5 (60–73)	
Autoimmune diseases^c^	17	6.8	35 (34–40)		31 (28–35)		67 (62.5–72)	
Renal failure	15	6	35 (32–36)		33 (29–35)		66 (62–71)	
Cancer	24	9.6	33.5 (32–35.75)		33 (28.25–35)		65.5 (61–69.75)	
Harmful behaviours			Mean (Q1–Q3)	*U*	Mean (Q1–Q3)	*U*	Mean (Q1–Q3)	*U*
Yes	43	17.3	36 (32–38)	4168	33 (28–35)	4001.5	67 (62–73)	4105
No	206	82.7	35 (32–38)	0.541	32 (28–35)	0.312	66.5 (61–71)	0.450
Receiving care			Mean (Q1–Q3)	*U*	Mean (Q1–Q3)	*U*	Mean (Q1–Q3)	*U*
Yes	172	69.1	35 (32–37)	5807.5	32 (28–35)	6547	67 (61–71)	6359.5
No	77	30.9	36 (32–39)	0.119	32 (28–35)	0.885	67 (60.5–71.5)	0.617

Abbreviations: KW, Kruskal–Wallis test; *U*, Mann–Whitney *U* test.

^a^ Asthma, COPD.

^b^ Coronary artery disease, arrhythmia, heart failure.

^c^ Psoriasis, rheumatoid arthritis, Crohn's disease.

A weak positive correlation was found between the age of the patients and the total score of family peace (*r* = 0.149; *p* = 0.019) (Table 4). As the level of self‐efficacy of the patients increased, it was found that their family peace increased significantly in a positive direction (*r* = 0.389, *p *< 0.001) (Table 4).

**Table 4:** Correlation distributions between age, duration of chronic disease, number of medications used and family peace sub‐dimension and total dimension scores of the individuals (*n* = 249).*

Variables		The deep wear	The general contentment	Total
Age (year)	**R**	0.155	0.101	0.149
	**P**	0.015	0.111	0.019
Chronic disease duration (month)	**R**	0.093	0.121	0.120
	**P**	0.144	0.057	0.058
Amount of medication used (piece/day)	**R**	−0.024	0.045	0.008
	**P**	0.702	0.476	0.902
Self‐efficacy scale score	**R**	0.281	0.414	0.389
	**P**	< 0.001	< 0.001	< 0.001

*Spearman's correlation test.

**Figure 2:**
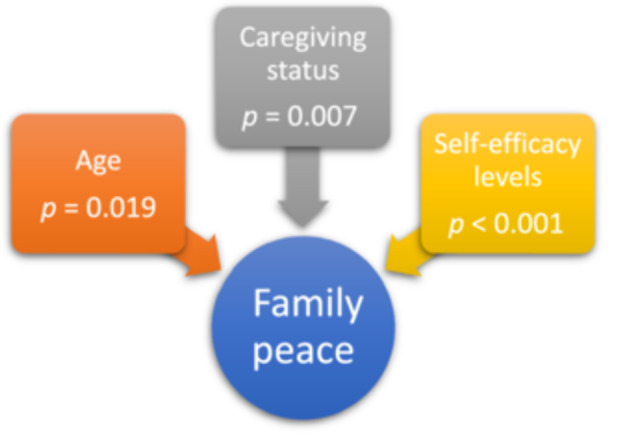
Situations affecting family peace in individuals over 65 years of age with chronic disease.

Family peace is found to be impacted by factors such as age, dependents and self‐efficacy in people over 65 who have chronic diseases (Figure 2).

## 4 Discussion

This study aimed to understand the level of family peace and the factors affecting family peace of individuals with advanced chronic illness. When interpreting the results of the study, it is important to explain that the study population represents a population of individuals with chronic diseases and self‐efficacy levels above the average, with an average age of 73 years, most of whom can meet their economic expenses and have a nuclear family structure. In our study, the family peace of elderly individuals was above average. As in the world, interest in successful ageing is increasing in our country. Successful ageing is considered as a complex combination of physiological, psychological, social and cultural factors [19]. In this context, although the concept of family peace within social factors–social resources is among the topics that need to be studied, especially in the context of health services, research on the family peace of elderly people with chronic diseases is limited. In the studies on social resources [7, 16, 17, 18, 19], it is seen that people such as family, friends, neighbours and care home workers are considered together, and family peace is not evaluated.

In our research, it was determined that the family peace of individuals improved with increasing age. Families go through small and large changes over time, including changes in who lives in the household through marriage, divorce, remarriage, birth or death, as well as changes in the health and care needs of family members, such as a child becoming a toddler, going to school, a parent going to work or being treated for a serious illness. The family life of a newly married couple or a couple with young children is different from the family life of an older couple with adult children or an elderly person with an illness [6]. It is therefore useful to assess the concept of well‐being according to the family life cycle. Increasing attachment to the family with increasing age and the tendency to avoid conflicts with family members may explain this positive relationship between age and family peace [23]. This finding of ours is consistent with Charles and Carstensen's findings that individuals become more selective in their social relationships with age and contribute to family peace by avoiding relationships that do not provide emotional satisfaction [24].

In our study, there is a strong positive relationship between family peace and self‐efficacy (*r* = 0.389, *p *< 0.001). For elderly parents, ‘doing the best’ and ‘maintaining dignity’ are themes related to aging, and these themes emerge in different ways depending on the structure of the family and the relationships of family members [6]. In addition, Bandura's self‐efficacy theory suggests that individuals' sense of self‐efficacy contributes to more harmonious family relationships by increasing their capacity to manage the factors that cause stress in their lives [25]. Studies have also shown that as the self‐efficacy levels of individuals with chronic diseases increase, their ability to cope with the disease and psychological well‐being increase and this positively affects family harmony [13, 26]. Elosua listed the sub‐dimensions related to quality of life in the elderly under five headings according to the degree of importance and reported that the first three of these headings were health, autonomy and maintaining family support, respectively [16]. Therefore, it is possible to say that the self‐efficacy level of elderly individuals with chronic diseases may affect family peace.

Individuals who provide care to their relatives have been found to experience lower levels of family peace than those who do not provide care (*p* = 0.004, *p* = 0.030 and *p* = 0.007, respectively, for the total score and two sub‐dimensions). Family caregivers reported that care responsibilities seriously compromised their psychological health, or at least that these responsibilities were associated with a very high level of stress [27], which likely is a contributor to a significant reduction in family peace. In one study, it was reported that as the symptom burden of patients increased, the family peace of caregivers was negatively affected [13]. In another study, it was reported that elderly family members concealed their own thoughts, feelings and needs in order to spend a positive last time with the patient in the last period of cancer, not to burden each other and to avoid tension, and that this strategy worked most of the time, but occasionally conflicts and tensions were experienced with different needs [27]. Altogether, these findings underscore not only the need to improve the self‐efficacy of family caregivers but also the need to attend to the elevated risk of compromised family peace associated with the prolonged period of caregiving. In this context, the results of our study suggest that social support programmes are needed to increase family peace in families with individuals in need of care.

The study's findings indicate that there was no statistically significant difference in the patients' family peace scale total and sub‐dimension scores based on factors such as gender, marital status, educational attainment, income level, type of chronic illness, presence of caregivers, or harmful habits (*p *> 0.05). This finding partly contradicts the result that older women are more likely to turn to family members than older men [28]. However, the fact that female and male participants did not differ statistically in terms of family peace in our study suggests that the search for support is not only related to gender but also to the effectiveness of social support mechanisms. In our study, in terms of marital status, although being married or single did not have a significant effect on family peace, it was observed that a large proportion of the elderly (69.1%) received care support; this is consistent with the findings of Antonucci and Akiyama that elderly individuals seek support not only from their spouses but also from their wider social environment [26].

In terms of family type, no significant difference was found between nuclear and extended family structures in our study. This result partially contradicts the findings of Pinquart and Sörensen, who stated that elderly individuals in need of care feel the burden of care more in nuclear families [29]. Zarit and colleagues state that extended family structures can provide social support that stabilises care‐related stress [30]. However, the findings observed in our study suggest that family type alone has a limited effect on determining family tranquility and that family support mechanisms may be more determinative.

## 5 Conclusion

This study evaluated the levels of family peace in individuals over 65 years of age with chronic disease and evaluated the relationships of these variables with socio‐demographic and medical factors. The findings of the study show that family peace is related to age, dependents and self‐efficacy perceptions of individuals. Individuals' feeling of self‐efficacy and being able to perform their daily activities independently both increase their personal satisfaction and positively affect their level of peace within the family. This finding suggests that social and rehabilitation programmes that develop self‐efficacy can provide significant improvements in the family relationships and overall quality of life of elderly individuals. In order to increase family peace, it is recommended that social support mechanisms should be strengthened and programmes that encourage individuals' sense of self‐efficacy should be disseminated. Focusing the social policies to be developed for elderly individuals on services that support intra‐family relationships and protect the independence of individuals can make significant contributions to improving the overall quality of life. In particular, the dissemination of home care services and self‐efficacy‐oriented rehabilitation programmes may positively affect both family peace and social welfare of individuals.

### 5.1 Limitations

The individuals in the study population are limited to a single health centre. Additionally, the cross‐sectional nature of the study design may prevent inferences about causality. Therefore, the results should not be generalised to the population.

## Conflicts of Interest

The authors declare no conflicts of interest.

## Data Availability

The data that support the findings of this study are available from the corresponding author upon reasonable request.
